# The six minute walk test in healthy young women

**DOI:** 10.1016/j.jsampl.2023.100052

**Published:** 2024-01-09

**Authors:** Madeleine G. Spicer, Alicia T. Dennis

**Affiliations:** Department of Anaesthesia, The Royal Women's Hospital, Corner Grattan St & Flemington Rd, Parkville, 3052, Australia

**Keywords:** Reference values, Walk test, Six minute walk test, Exercise test, Female, Women

## Abstract

**Objectives:**

The Six Minute Walk Test (6MWT) assesses functional capacity. We determined the reference range distance walked, heart rate change, and breathlessness in healthy women.

**Design:**

Prospective observational study.

**Methods:**

100 nulliparous women aged 18–40 performed two 6MWTs, with the second performed after heart rate returned to baseline. Borg scales compared expected and true breathlessness.

**Results:**

Reference range was 472–758 ​m, with distance mean (SD) of 615 (73.0) metres. Heart rate increase during exercise was 11 (11.8) beats per minute, recovering to baseline in 4.5 (3.9) minutes. 63 ​% of women overestimated breathlessness.

**Conclusions:**

These data represent reference values for functional evaluation of healthy young women.

## 1 Introduction

Functional walking tests are commonly used due to ease, affordability, and minimal equipment requirements. The Six Minute Walk Test (6MWT) measures submaximal exertion which is a good predictor of true functional status, and is the best-validated walk test for use across populations [1].

Reference values for older, comorbid patients are well-characterised, but comparable data in young, healthy women lacks due to small cohort sizes and pooling of studies of men and women. The 6MWT may feasibly risk-stratify women in maternity and perioperative contexts, especially in lower-resource settings, as it accurately predicts maximal oxygen consumption (including in healthy adults) [1,2]. Increasing rates of metabolic disease and sedentary lifestyle suggest the 6MWT as a useful functional assessment, and the characterisation of a non-pregnant control group is necessary as an extension of previous pregnancy-related exercise studies [3].

## 2 Methods

After Human Research Ethics Committee approval (Royal Women's Hospital HREC (EC 00259), Project 15/23) and Australian and New Zealand Clinical Trials Registry registration (ACTRN: 12615000964516), 100 female volunteers were recruited over six months by flyers posted at local hospitals and universities. Inclusion criteria were nulliparous females aged 18–40 years, conforming with American Society of Anesthesiologists (ASA) Classification I or II (healthy individuals or mild systemic disease). Exclusion criteria included BMI >30 ​kg/m^2^ and tobacco use.

After giving written informed consent, participants underwent the 6MWT according to guidelines in a 30-m corridor [4,5]. After 5 ​min of rest, blood pressure (BP), heart rate (HR), blood oxygen saturation levels (SpO_2_) and respiratory rate (RR) were recorded using a calibrated automated observations device (Welch Allyn Connex Spot Monitor, 15 ​V). Participants were instructed to “walk as far as possible for 6 ​min” and received standardised encouragements minutely.

After completion, initial observations were collected at 1-min post-exercise. To standardise timing, 1 ​min was chosen as the practical time point to move from walking to seated. Then BP, HR, and SpO_2_ readings were taken minutely, and RR readings five-minutely [6], until HR returned to within four beats per minute (bpm) of resting level (taking minimum five and maximum 15 ​min). Whilst many studies use time or rate of decrease per minute to define recovery from exercise, our limit of 4 bpm from original baseline was chosen to reflect normal fluctuation of baseline HR, factoring in the effects of exercise on short-term and ultra-short-term HR variability.

A further 6MWT was then conducted after the participants’ HR returned to baseline. The Borg Rating of Perceived Exertion (RPE) Category Scale and the Modified Borg Dyspnoea (MBD) Scale questionnaires were administered after the second 6MWT [7], to assess true and expected levels of effort, and true and expected levels of breathlessness respectively.

SPSS Statistics Version 24 (IBM© SPSS© Statistics Version 24 IBM Corporation 2016, Chicago, Ill) was used for data analysis. The distribution of data was assessed using Kolmogorov–Smirnov and Shapiro–Wilk tests for normality. Normally distributed data were compared using parametric tests and non-normally distributed data were compared using non-parametric tests.

The 95 ​% reference interval for the 6MWD was calculated using mean distance walked ±1.96SD. The 95 ​% confidence intervals (CI) of the reference interval and resting HR were calculated using the standard error of the limit of the reference interval such that a 95 ​% CI for the lower and upper limits were determined (https://www-users.york.ac.uk/∼mb55/intro/refint.htm).

The relationships between resting HR and distance walked, and change in HR with exercise and rate of HR recovery, were explored using linear regression and correlation. The 95 ​% CI for the line of best fit was calculated, and Pearson r with its associated p value was determined.

Expected and actual exertion and breathlessness data were analysed using the Wilcoxon matched-pairs signed rank test. Two-sided p values are given, with a significance level alpha of 0.05.

## 3 Results

One hundred and one participants were consented (with one excluded due to BMI). Participant characteristics and resting variables of the 100 women are shown in Table 1.

**Table 1:** Participant characteristics and resting physiological variables.

Variable	Mean (Standard Deviation) or Range
Age (years)	24.4 (3.3)
Height (m)	1.65 (0.07)
Weight (kg)	63.3 (10.7)
BMI (kg/m^2^)	23.2 (3.1)
Resting HR (bpm)	71.1 (10.8)
Reference Interval for Resting HR (bpm)	50–92
95 ​% CI lower limit, upper limit (bpm)	48 to 52, 90 to 94
Resting Systolic BP (mmHg)	112.4 (9.3)
Resting Diastolic BP (mmHg)	74.1 (7.1)
Resting SpO_2_ (%)	98.5 (1.0)
Resting RR (brpm)	14.3 (3.8)

BMI: Body Mass Index; HR: heart rate; BP: blood pressure; SpO_2_: blood oxygen saturation; RR: respiratory rate.

The average (SD) distance walked during the first 6MWT was 605.9 (72.1) metres, and during the second was 623.9 (75.8) metres – an average difference of 18 ​m. 86 ​% of participants walked farther on their second attempt. Overall, the average 6MWD was 614.9 (73.0) metres, with a reference range of 472–758 ​m (95 ​% CI lower limit 458–486 ​m; 95 ​% CI upper limit 744–772 ​m). Average walking speed was 6.2 (0.7) km/hr.

Mean HR increase with exercise was 11 bpm, from an average resting value of 71 bpm to 82.5 bpm at 1 ​min post-exercise on the first attempt (Table 2). Mean HR 1 ​min post-second 6MWT was higher than post-first (mean difference 4 bpm, 95 ​% CI 2–5 bpm, p ​< ​0.0001). Whilst average HR recovery was 4 ​min, women demonstrated a wide variety of recovery times.

**Table 2:** Post-exercise variables.

	Variable	Mean (Standard Deviation)
6MWT One	HR at 1 ​min post-exercise (bpm)	82.5 (17.4)
	Mean HR increase with exercise (bpm)	11.4 (11.8)
	Time to recovery (HR within four beats of resting) (mins)	4.0 (3.5)
6MWT Two	HR at 1 ​min post-exercise (bpm)	86.4 (19.3)
	Mean HR increase with exercise (bpm)	15.3 (14.6)
	Time to recovery (HR within four beats of resting) (mins)	4.5 (3.9)
Mean Data at One Minute	HR peak (bpm)	110 (23.5) (95 ​% CI 106–114)
	SBP peak (mmHg)	130 (14.8) (95 ​% CI 127–133)
	DBP peak (mmHg)	80 (7.5) (95 ​% CI 76–83)
	RR peak (brpm)	19 (4.6) (95 ​% CI 18–20)
	SpO 2 peak (%)	99 (1.3) (95 ​% CI 98–99)

	Variable	Median (IQR)
Mean Borg Data	Mean MBD score (1–10)	5 (3,7)
	Mean RPE score (1–15)	0.5 (0,1)
	Variable	Proportion

	Proportion of women whose expectation of breathlessness was greater than experienced breathlessness	62 ​%
Proportion of women whose expectation of exertion was greater than experienced exertion	32 ​%

6MWT: Six Minute Walk Test; mins: minutes; HR: heart rate; bpm: beats per minute; SBP: systolic blood pressure; DBP: diastolic blood pressure; mmHg: millimetres of Mercury; RR: respiratory rate; brpm: breaths per minute; SpO 2: peripheral oxygen saturation; CI: confidence interval; IQR: interquartile range; MBD: Modified Borg Dyspnoea; RPE: Rating of Perceived Exertion.

**Fig. 1:**
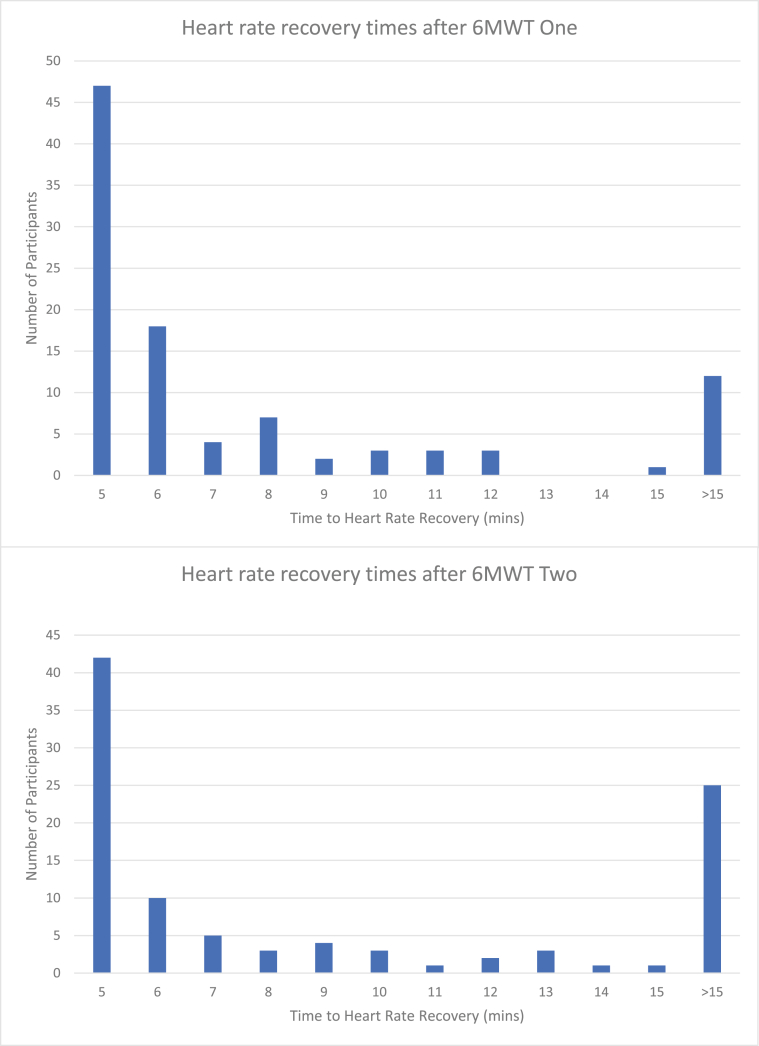
Heart rate recovery times after Six Minute Walk Test One and Two.

Heart rate recovery after the first 6MWT occurred in sixty-two of 100 women (62 ​%) within 5 ​min, 19 of 100 (19 ​%) within 10 ​min, and seven of 100 (7 ​%) within 15 ​min. Twelve of the 100 women (12 ​%) did not recover within the 15-min timeframe. Heart rate recovery was slower after the second 6MWT: fifty-two (52 ​%) recovered in 5 ​min, 16 (16 ​%) took 10 ​min, 8 (8 ​%) took 15 ​min, and 24 (24 ​%) did not recover within 15 ​min (Fig. 1).

There was no evidence that a lower resting HR was associated with increased distance walked (r ​= ​0.001, 95 ​% CI -1.342–1.327, p ​= ​0.992). However, women experiencing a greater HR change with exercise had prolonged recovery time (r ​= ​0.712, 95 ​% CI 0.591–0.802, p ​< ​0.0001).

Overall there was little difference between expected and actual exertion levels (p ​= ​0.590) as assessed by the Borg RPE scale. However, women expected to experience more breathlessness than they truly did, as assessed by the MBD scale (median difference 0.5, p ​< ​0.0001). Eight of 99 (8 ​%) and 19 of 99 (19 ​%) of women had no expectations for their level of exertion or breathlessness respectively, but of the women who did have expectations, 29 of 90 (32 ​%) anticipated greater exertion than they actually experienced, and 49 of 79 (62 ​%) overestimated their level of breathlessness.

## 4 Discussion

This study is one of the first to investigate the average 6MWD, cardiorespiratory response profile, and expectation of breathlessness and exertion associated with the 6MWT in young healthy females, with an aim to develop a control study to contrast to studies performed during pregnancy [3].

The reference range developed (472–758 ​m) reflects work by Kim et al. [8] (reference range 447–761 ​m, including male and female Korean participants), whose average 6MWD finding of 580.9 (47.8) metres in the female population (mean age 37.7 years) is consistent with our findings (average 6MWD of 614.9 (73) metres, mean age 24.4 years). Our increased 6MWD likely reflects a taller Australian population, with height influencing 6MWD [9]; average study heights respectively were 1.59 (0.05) versus 1.65 (0.07) metres. The mean age difference of 13.3 years is unlikely to contribute, given the effect of age is not significant until 60 years [10].

A broad 6MWD reference range is consistent throughout the literature [10]. Experimental design reduces variability by performing two 6MWTs (to diminish the ‘learning effect’); however, 86 ​% of participants still walked farther on their second attempt.

Overall, participants demonstrated good tolerance of the submaximal exercise challenge, as expected for a young, healthy cohort. Halliday et al. posit that heart rate response may be a useful marker of effort in healthy candidates [11]; in this study, the average (SD) time to HR recovery was 4.5 (3.9) minutes after the second 6MWT, compared to 4.0 (3.5) minutes after the first. Mean HR increase at 1 ​min post-exercise was on average 3.9 bpm higher after the second attempt. The effect of repeated exertion was seen, with twice the number of women failing to reach heart rate recovery after the second 6MWT compared to the first; however, greater exertion during the second 6MWT is likely, given the further distance covered on average.

These 6MWT results may facilitate identification of women whose heart rate patterns do not fit the expected responses. Using a validated formula for maximal HR (HR_max_) prediction by Shargal et al. [12], HR_max_ for our cohort with average age 24.4 is 189.7 bpm. As such, the percentage HR_max_ after the first 6MWT was 43.5 ​%, and after the second was 45.5 ​%. Whilst Shargal et al. did find that HR_max_ values decrease faster with age in women than men [12], Nes et al. [13] reported that older equations underestimated HR_max_ in subjects over 30 years, and only minor age-adjusted differences in age cohorts existed; as such, it is expected that the upper age limit of our cohort would respond similarly to the lower limit in terms of HR_max_. Practically, HR increase which is disproportionate to the submaximal exercise challenge (especially after the second attempt, given the increment of 2 ​% of HR_max_ seen on repetition), may demarcate those at increased risk. Notably though, HR responses to exercise were a secondary outcome, with our primary outcome being elucidating 6MWD in order to compare to values obtained in pregnancy.

Breathlessness assessment also assists with developing a control group to data in healthy pregnant people. Many women overestimated their expected level of breathlessness compared to their actual experience, indicating the 6MWT is a tolerable submaximal exercise test. However, most women did find the test more physically challenging than anticipated, with two in three women expecting lesser exertion than they experienced. This is likely attributable to the perceived ‘ease’ of walking, found more effortful as women focus on exerting themselves. As such, utilising the perceived difficulty of the test to differentiate at-risk women is not reliable.

Study limitations include potential engagement in vigorous walking or cycling by participants prior to testing, which may have impacted findings. Additionally, as participant motivation influences results, outcomes do not necessarily reflect maximal efforts of this population. There was considerable variability in effort level, which decreases standardisation; however, this is an inherent feature of the 6MWT. Furthermore, a limitation to interpretation is the assumption that HR increases are greater than reported as initial monitoring occurred at the 1-min post-exercise mark. As such, true peak submaximal heart rate was likely not recorded; however, delayed decrease in heart rate during the first minute after exercise, a potential marker of decreased vagal activity, is a predictor of mortality, and thus elevated HR at 1 ​min is likely of significance [14].

## 5 Conclusion

With these findings of an average (SD) 6MWD of 614.9 (73.0) metres (reference range 472–758 ​m), and mean HR changes of 11.4 (11.8) and 15.3 (14.6) bpm after the first and second 6MWT respectively, health assessment of the young female population can be facilitated. The Six Minute Walk Test is easily implementable, acceptable to the participant, and provides important information about submaximal exercise capacity.

## Ethics approval

After Human Research Ethics Committee approval (Royal Women’s Hospital HREC (EC 00259), Project 15/23) and registration with the Australian and New Zealand Clinical Trials Registry (ACTRN: 12615000964516), participants gave written informed consent. This trial was conducted in line with accepted ethical practices.

## Financial disclosures

Funded by an Australian and New Zealand College of Anaesthetists Academic Enhancement Grant 2018, and Fulbright Future Scholar funding with courtesy of the Kinghorn Foundation.

## Declaration of competing interest

None.
